# Long-term outcomes after non-aneurysmal, non-traumatic subarachnoid hemorrhage: a prospective multicenter outcome study

**DOI:** 10.1007/s00415-026-13939-2

**Published:** 2026-06-25

**Authors:** Wouter J. Dronkers, Femke Geelen, Alyson Gross, René van den Berg, Jonathan M. Coutinho, Luca Regli, W. Peter Vandertop, Dagmar Verbaan, Menno R. Germans

**Affiliations:** 1 Department of Neurosurgery, Amsterdam UMC location University of Amsterdam, Meibergdreef 9, room, H2-259, 1105 AZ Amsterdam, The Netherlands; 2 Neurovascular Disease, Amsterdam Neurosciences, Meibergdreef 9, Amsterdam, The Netherlands; 3 Department of Radiology and Nuclear Medicine, Amsterdam UMC location University of Amsterdam, Meibergdreef 9, Amsterdam, The Netherlands; 4 Department of Neurosurgery, University Hospital Zurich, University of Zurich, Zurich, Switzerland; 5 Clinical Neuroscience Centre, Zurich, Switzerland; 6 Department of Neurology, Amsterdam UMC location University of Amsterdam, Meibergdreef 9, Amsterdam, The Netherlands

**Keywords:** Subarachnoid hemorrhage, Outcome, Return to work

## Abstract

**Objective:**

Non-aneurysmal, non-traumatic subarachnoid hemorrhage (nSAH) refers to cases where a causative aneurysm cannot be identified. Stratification into perimesencephalic non-aneurysmal SAH (PMSAH) and non-perimesencephalic non-aneurysmal subarachnoid hemorrhage (NPSAH) is important for clinical purposes. The nSAH patients, in particular PMSAH patients, tend to have a better prognosis, as compared with aneurysmal subarachnoid hemorrhage patients. We prospectively studied functional outcome, return to work (RTW) status, quality of life and residual symptoms at least one year after the hemorrhage in these patients.

**Methods:**

This multicenter study, conducted in Amsterdam UMC (Netherlands) and University Hospital Zurich (Switzerland), included adult nSAH patients admitted between 2015 and 2021. Outcomes, including the modified Rankin Scale (mRS), RTW status, the EuroQol-5Dimensions, EuroQol-Visual Analogue Scale, the Checklist Cognition and Emotion 24 items, and the Stroke-Specific Quality of Life Scale were prospectively assessed.

**Results:**

Of 262 potential participants, 111 patients (66 NPSAH and 45 PMSAH; response rate 42%) returned the questionnaire, whereas 109 (41%) patients completed the telephone interview. The median follow-up time was 58.2 months (range 28.9–72.3). An mRS-score of ≥2 was recorded in 44 (40%) patients. Among 85 of 87 (RTW status missing in two patients) previously employed patients at the time of the hemorrhage, 61 (70%) fully RTW, 11 (13%) partially RTW, and 13 (15%) did not return to work. Twenty-eight (26%) patients had no complaints at all at follow-up. Frequently reported residual symptoms involved increased tiredness and difficulties remembering new information.

**Conclusions:**

Long-term follow-up of nSAH patients reveals that 40% reports an mRS-score of ≥2. Furthermore, 28% of previously employed patients did not (fully) return to work and the majority of patients reports residual symptoms at follow-up. Our findings show that a larger part of non-aneurysmal, non-traumatic SAH patients can not resume all previous activities than previously reported, even at long-term follow-up.

**Supplementary Information:**

The online version contains supplementary material available at 10.1007/s00415-026-13939-2.

## Introduction

A non-traumatic subarachnoid hemorrhage (SAH) is a condition with high morbidity and case fatality [1]. In approximately 15% of SAH patients, no aneurysmal cause for the hemorrhage is found. Compared to aneurysmal SAH (aSAH) patients, those with non-aneurysmal SAH (nSAH) generally have better outcome regarding mortality and functional recovery [2].

Outcome assessment in nSAH patients has predominantly been done within the first 3–12 months after the hemorrhage [2–10]. Studies reporting on outcomes at least one year after the hemorrhage have mostly been done through retrospective analyses [11–17], or included a small prospective sample [18–20]. Moreover, most of these studies focused on functional outcome, while fewer have assessed the quality of life and return to work (RTW) status [18, 21–25].

This study aimed to prospectively assess the functional outcome, RTW status, residual symptoms, and quality of life in nSAH patients at long term follow-up.

## Methods

### Patient population and diagnostic work-up

A multicenter study was conducted in the Neurosurgery Departments at the Amsterdam University Medical Centers (AUMC) in the Netherlands and University Hospital Zurich (USZ) in Switzerland, both tertiary referral centers for the treatment for all SAH patients in the referral area. Consecutive adult nSAH patients, admitted to either center between January 1 st 2015, and April 1 st 2021 were invited to participate in this study. SAH was diagnosed according to current international standards [26]. Exclusion criteria included: patients with an identifiable cause of SAH (e.g. traumatic, aneurysmal or due to other intracranial vascular pathology); patients unable to read and write in general; patients unable to read, comprehend, write or speak in Dutch or English (AUMC) or German or English (USZ); patients who did not provide consent, and non-responders to the invitation. Additionally, patients who relocated abroad were excluded.

In accordance to previous research, nSAH was stratified into perimesencephalic non-aneurysmal SAH (PMSAH), as defined by van Gijn et al. [27], and non-perimesencephalic non-aneurysmal subarachnoid hemorrhage (NPSAH). All nSAH patients who did not meet the PMSAH criteria, including CT negative, lumbar puncture (LP)-positive SAH, were classified as NPSAH. Patients were treated in accordance with current international guidelines for SAH management [26]. For diagnostic work-up, all nSAH patients underwent at least a non-contrast CT-scan and CT-angiography. Digital subtraction angiography (DSA) was not routinely performed in PMSAH patients. For non-perimesencephalic, DSA-negative cases, follow-up imaging (DSA, CTA, and/or MRI/MRA) was typically conducted 10–14 days post-hemorrhage, with exceptions determined during interdisciplinary neurovascular board reviews. NPSAH patients received two to three weeks of nimodipine. PMSAH did not receive nimodipine. Institutional Board Approval was granted from the Medical Ethics Committee of the AUMC (W22_107) and from the Ethics Committee of the USZ (BASEC nr. 2022-00732), separately, prior to the recruitment of eligible patients.

### Study design

Patients who met the inclusion criteria received the patient information folder, an informed consent form, and three questionnaires assessing quality of life per physical mail in Dutch or German, according to the hospital in which they were treated. Patients were invited to participate in the study, sign the informed consent form, and complete the questionnaires. A reminder was sent to all non-responding patients within two months after the initial invitation. Patients who returned their signed informed consent were scheduled for a telephone interview. Patients were asked in the information folder to seek help (e.g., a caregiver or relative) for filling out the questionnaires or during the telephone interview if needed. No compensation or payment was provided for participation in the study.

### Data collection

Demographic data of all consecutive patients with nSAH as well as their functional outcome were prospectively registered in both hospitals. Additional relevant clinical data collection was conducted prospectively at the AUMC and by retrospective chart review at the USZ. Demographic characteristics collected included: sex, age, history of smoking, Glasgow Coma Scale (GCS) score, World Federation of Neurosurgical Societies (WFNS) score at time of admission to the treatment center, and Fisher grade, scored by the treating physician. Relevant medical history included intracranial hemorrhage (hemorrhagic cerebrovascular accidents, traumatic hemorrhages), hypertension, and cardiovascular disease (ischemic cerebrovascular accident, cardiac arrest and other cardiac and vascular pathologies). Relevant medication involved the use of anticoagulant and/or antiplatelet medication. Clinical characteristics that were collected involved in-hospital mortality, complications (recurrent bleeding [rebleeding], delayed cerebral ischemia [DCI], and hydrocephalus) and whether patients received some form of rehabilitation. The structured telephone interviews were conducted by two trained research scholars (author FG for the AUMC and author AG for the USZ), both of whom were Dutch or German native speakers, respectively, and proficient in English language. All results were reported according to the STROBE guidelines.

### Objectives and outcome measures

The objectives of the study were prospectively assessing functional outcome, RTW status, presence of residual symptoms, and quality of life (QoL) at least one year after the hemorrhage. Functional outcome and return-to-work rates were both assessed using the telephone-administered, validated version of the modified Rankin Scale (mRS, score 0–6) [28, 29]. A lower mRS score indicates a better functional outcome. Residual symptoms were assessed using the Checklist Cognition and Emotion 24 items (CLCE-24; 22 yes/no and 2 open items, score 0–24) [30]. A lower score indicates fewer residual symptoms with a score of ‘0’ indicating no symptoms. Qol was assessed with the Stroke-Specific Quality of Life Scale (SSQoL; 49 items with a 5-point Likert scale, score 49–245) [31], and the EuroQol-5Dimensions (EQ-5D-5L; five items with a 5-point Likert scale, score 0–1,000) and EuroQol-Visual Analogue Scale (EQ-VAS; one item, score 0–100) [32]. Dutch reference scores were used for both centers since to date, no Swiss reference scores exist. For the SSQoL, scores are divided by the number of items per domain, resulting in a minimum score of 1 and maximum score of 5. For the SSQol, EQ-5D-5L, and the EQ-VAS, higher scores reflect better quality of life.

### Statistical analysis

Descriptive statistics were reported for all baseline characteristics, clinical course characteristics, and outcomes for nSAH patients, and stratified per diagnosis (NPSAH and PMSAH). Continuous variables were tested for normal distribution, with a Shapiro-Wilk value >0.9 defined as normally distributed. Normally distributed data were reported as means with standard deviations (SD) and non-normally distributed data as medians with interquartile range (IQR), respectively. Student’s t-test was done for normally distributed continuous variables and Mann-Whitney U test for non-normally distributed continuous variables, respectively. Dichotomous and categorical variables, as well as outcomes, were compared using a Chi-square test. Functional outcome was dichotomized in mRS-score 0–1 and mRS-score 2–6. Additionally, an analysis was performed using mRS-score 0–2 and mRS-score 3–6. RTW status was dichotomized into ‘fully returned to work’ and ‘partially (other work or lower workload) or not returned to work’. Stay at home, retired or incapacitated patients were excluded from this analysis. Quality of life scores were reported as total mean (SD) or median (interquartile range [IQR]) scores and per subscale if applicable. Outcomes were reported for the total nSAH group. Furthermore, outcomes were compared between NPSAH and PMSAH. Baseline characteristics and the clinical course were compared between responders and non-responders. Results with a *P* value <0.05 were considered statistically significant. All analyses were done using SPSS statistics, version 28.0.

## Results

### Recruitment and patient characteristics

**Fig. 1:**
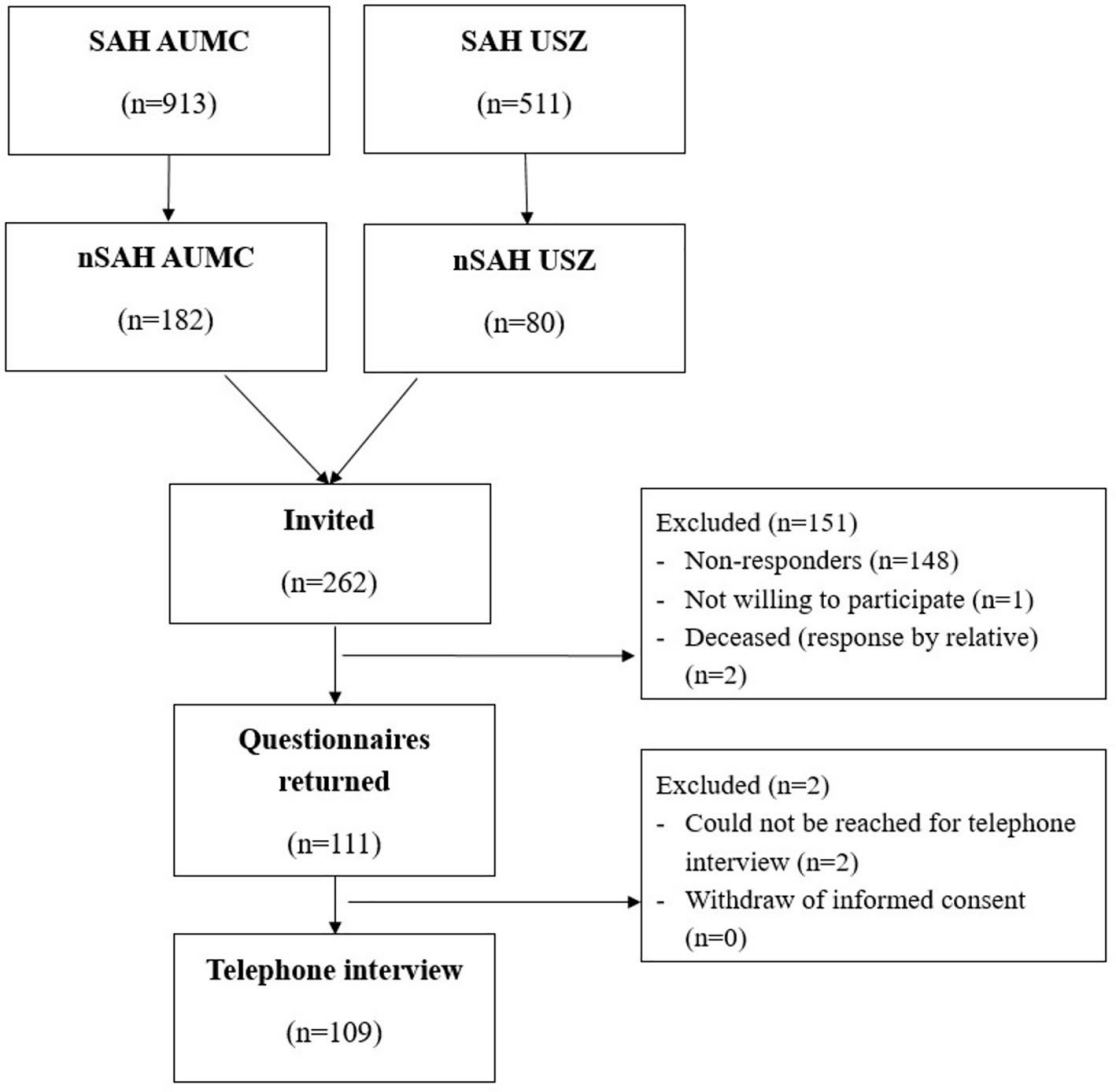
Flowchart of included patients

Between 2015 and 2021, 1424 non-traumatic SAH patients were admitted to our facilities (913 AUMC and 511 USZ). Five nSAH patients, all NPSAH, died during the clinical course. A total of 262 nSAH patients (182 center AUMC and 80 center USZ) were invited to participate in the present study (Fig. 1). Of these, 111 patients (66 [59%] NPSAH, 45 [41%] PMSAH; 43 [39%] female; mean age [SD] 55.6 years [9.6]) provided consent and returned their completed questionnaires, resulting in a response rate of 42% (Table 1). One hundred and nine (41%) patients participated in a telephone interview. The majority of the included patients (*n*=87; 78%) were employed at the time of hemorrhage. Acute hydrocephalus was reported in 25 (23%) patients. After discharge from the treatment center, 50 (45%) patients received some form of rehabilitation. Cardiovascular disease and employment status differed between responders and non-responders (supplement table 1). Between centers, a difference in smoking status was reported (supplement table 2).

**Table 1:** Baseline characteristics and clinical course of 111 nSAH patients

	NPSAH *n*=66	PMSAH *n*=45	Total *n*=111	*p*-value
Age, years, mean (SD)	57.7 (9.6)	52.5 (8.6)	55. 6 (9.6)	0.005
Sex, female	30 (46)	13 (29)	43 (39)	0.079
Medical history				
Hypertension	19 (29)	8 (18)	27 (24)	0.184
Cardiovascular disease	8 (12)	2 (4)	10 (9)	0.165
Intracranial hemorrhage	1 (2)	0 (-)	1 (1)	0.407
Anticoagulation/platelet therapy	8 (12)	2 (4)	10 (9)	0.165
Smoking	30 (46)	17 (38)	47 (42)	0.422
Employment status				
Employed	47 (71)	40 (89)	87 (78)	0.026
Unemployed	0 (-)	0 (-)	0 (-)	Na
Not-applicable*	19 (29)	5 (11)	24 (22)	Na
Admission characteristics				
WFNS grade I-III	63 (96)	45 (100)	108 (97)	0.147
Complications				
Acute hydrocephalus	22 (33)	3 (7)	25 (23)	<.001
Recurrent bleeding	0 (-)	0 (-)	0 (-)	Na
Delayed cerebral ischemia	3 (5)	0 (-)	3 (3)	Na
Rehabilitation				
Rehabilitation (any form)	38 (58)	12 (27)	50 (45)	0.001

Provides an overview and comparison of baseline demographics and clinical course characteristics between responders and non-responders. Data is presented as numbers (%) unless otherwise specified

*Including retired, stay-at-home, and incapacitated patients

*nSAH* non-aneurysmal subarachnoid hemorrhage, *NPSAH* non-aneurysmal non-perimesencephalic subarachnoid hemorrhage, *PMSAH* perimesencephalic non-aneurysmal subarachnoid hemorrhage, *SD* standard deviation, *Na* not-applicable, *WFNS* World Federation of neurosurgical societies

**Table 2:** Outcome

	NPSAH *n*=66	PMSAH *n*=45	Total *n*=111	*p*-value
Follow-up time, median (IQR), months	60.1 (28.2–73.5	55.7 (31.3–71.2)	58.2 (28.9–72.3)	0.867
Functional outcome				
Physical dependency*				
Favorable outcome (mRS 0–2)	59 (91)	42 (96)	101 (93)	0.357
Excellent outcome (mRS 0–1)	40 (62)	25 (57)	65 (60)	0.622
RTW rate^†^				
Returned to work	40 (87)	32 (82)	72 (85)	0.531
Fully returned to work	35 (76)	26 (67)	61 (72)	0.347
Partially returned to work	5 (11)	6 (15)	11 (13)	0.747
Not returned to work	6 (13)	7 (18)	13 (15)	0.560
Quality of life				
EuroQol-5D-5L, mean score (SD)^‡^	0.857 (0.158)	0.868 (0.168)	0.861 (0.161)	0.730
EuroQol-VAS, mean score (SD)^‡^	76 (19)	78 (16)	77 (18)	0.726
CLCE-24, median (IQR) score	6 (1–10)	4 (0–11)	6 (0–11)	0.641
No problems	14 (21)	14 (31)	28 (25)	
Cognitive domain (1 or more complaints)	46 (70)	24 (53)	70 (63)	
Median (IQR) score, (range 0–13)	3 (0–6)	1 (0–6)	2 (2–6)	0.248
Emotional domain (1 or more complaints)	48 (73)	30 (67)	78 (70)	
Median (IQR) score, (range 0–9)	2 (0–4)	2 (0–4)	2 (0–4)	0.847
SSQoL^§^, median (IQR)				
Total score, median	4.64 (0.93)	4.73 (1.29)	4.71 (1.03)	0.784
Physical domain	4.78 (0.55)	4.89 (0.67)	4.85 (0.61)	0.287
Psychosocial domain	4.41 (1.09)	4.55 (2.07)	4.45 (1.45)	0.926

Data is presented as numbers (%) unless otherwise specified

^*^Calculated over 109 patients (65 NPSAH, 44 PMSAH)

^†^RTW return to work; calculated over 85 patients (46 NPSAH, 39 PMSAH)

^‡^Calculated over 110 patients (65 NPSAH, 45 PMSAH)

^§^Calculated over 109 patients (64 NPSAH, 45 PMSAH)

### Functional outcome and return to work

**Fig. 2:**
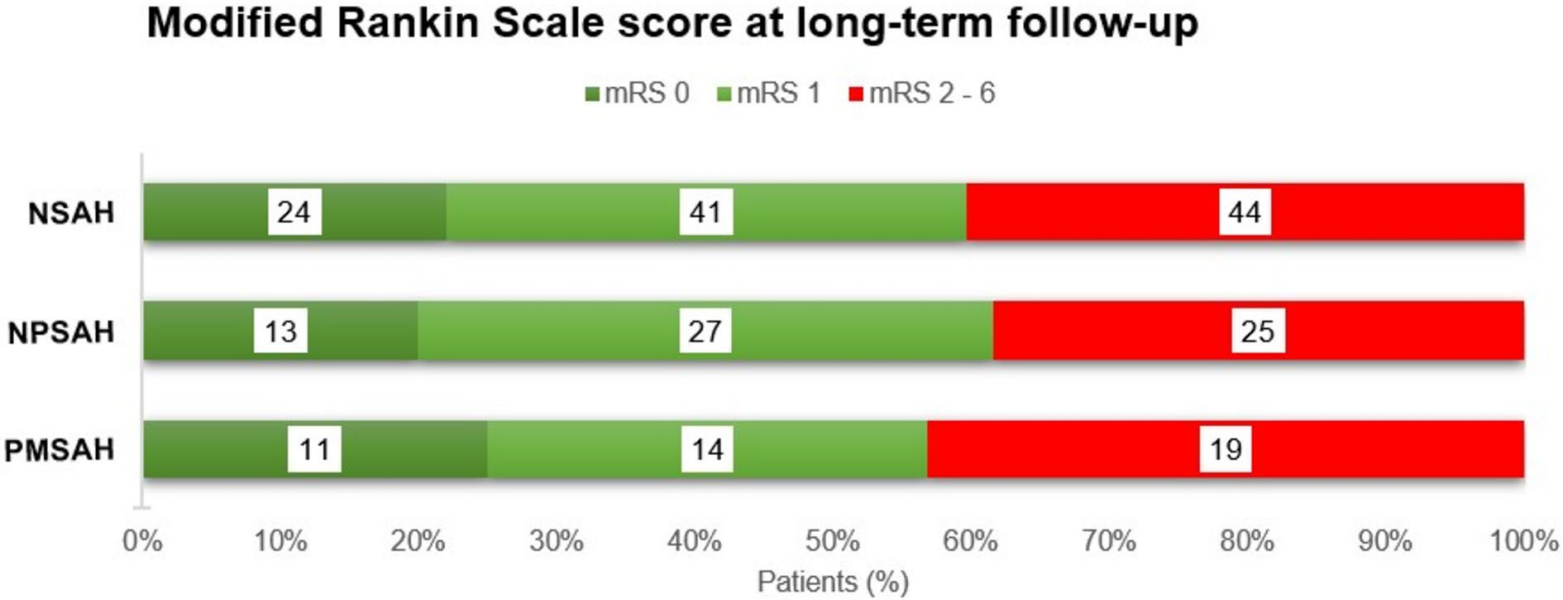
Modified Rankin Scale scores at long-term follow-up for the total number of patients and stratified per hemorrhage pattern. The numbers in the bar represent absolute number of patients

**Fig. 3:**
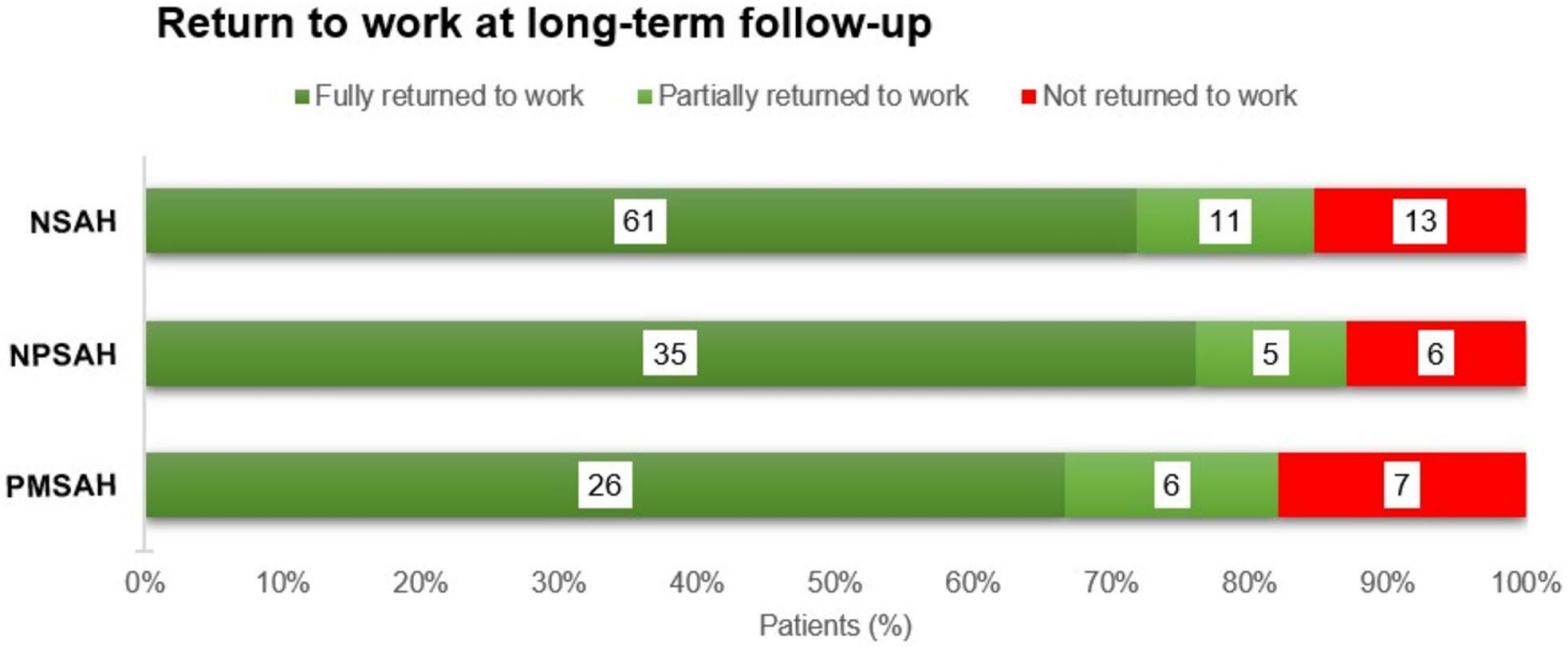
Return to work rates at long-term follow-up. The numbers in the bar represent absolute number of patients

The median (IQR) follow-up time between the hemorrhage and the telephone interview was 58.2 months (28.9–72.3 months) (Table 2). Forty-four (40%) patients scored a mRS score ≥2, indicating that not all previous activities were resumed (Fig. 2). Eight (7%) patients scored a mRS score 3, requiring some form of assistance in all-day life. Return to work status could be assessed in 85 of 87 patients that were previously employed. At follow-up, 24 (28%) did not (fully) return to work at follow-up (11 [13%] patients partially returned to work and 13 [15%] patients did not return to work at all) (Fig. 3).

### Residual symptoms and quality of life

Outcomes on the CLCE-24, assessed in 109 patients revealed that 81 (74%) patients reported cognitive and/or emotional residual symptoms at follow-up (median [IQR] number of complaints 6 [11]) (Table 2). Twenty-six (26%) reported no symptoms. Frequently reported symptoms were: increased tiredness (*n*=59; 53%), difficulties remembering new information (*n*=54; 49%), being more irritable (*n*=51; 46%), decreased attention (*n*=47; 42%), and difficulty keeping up or becoming slower (*n*=46; 41%) (Supplement Table 3). The SSQoL, the median (IQR) total, physical, and psychosocial scores were 4.7 (1.0), 4.9 (0.6), and 4.9 (1.5), respectively. Domains with lower median [IQR] scores were thinking (4.00 [2.33]), social roles (4.6 [2.0]), energy (4.7 [2.0]), and personality (4.7 [1.8]) (Supplement Table 4). The mean (SD) EQ-5D-5L and EQ-VAS scores were 0.861 (0.161) and 77 (18), respectively.

### NPSAH and PMSAH subgroup analysis

NPSAH patients were older and less likely to be employed compared to PMSAH patients (Table 1). Acute hydrocephalus was mostly reported in NPSAH (*p*<.001). Furthermore, NPSAH patients were more likely to be referred for rehabilitation (*p*=.001). An mRS-score 0–1 was reported in 40 (62%) NPSAH and 25 (57%) PMSAH patients (*p*=.470) (Table 2). RTW rates, presence of residual symptoms, or QoL scores rates were not significantly different between the groups.

## Discussion

This multicenter, observational study of non-aneurysmal, non-traumatic subarachnoid hemorrhage patients shows that 40% of living patients reported an mRS-score of 2 or higher at least one year after the hemorrhage, indicating that not all previously performed activities were resumed. Furthermore, 28% of patients did not fully return to work or did not return to work at all. Residual symptoms are frequently reported with only a quarter of patients denying symptoms. Therefore, the assumed ‘favorable functional outcome’ in these patients may be lower than previously reported.

Functional outcome in nSAH is often regarded as ‘favorable’ or even ‘excellent’, typically defined as mRS 0–2 and mRS 0–1, respectively [2, 3, 5, 6, 18, 33, 34]. However, outcome assessment methods differ markedly across studies, some rely on structured (telephone) interviews, others on retrospective chart reviews, introducing considerable variability and bias. Clinicians tend to assign lower (i.e. better) mRS scores, particularly within the 0–2 range, compared with patient-reported or structured interview-derived assessments [35, 36]. In our cohort we used a standardized telephone interview to mitigate this bias. Given that prior studies report the majority of patients achieving an mRS 0–2 within the first six months, reflecting slight disability (independent but not fully back to previous activities), our analysis specifically examined patients with mRS ≥2 and evaluated whether the proportion attaining an mRS 0–1 increased at the one-year mark post-hemorrhage. In the present study, 40% of patients still reported an mRS-score ≥2. Moreover, the proportion achieving an mRS-score 0–1 was noticeably lower than reported in previous nSAH series [5, 33, 34], which have reported favorable outcome rates ranging from 80 to 89% at follow-up intervals of six months to three years [5, 19]. Our findings are more in line with a previous study on outcome after aSAH, in which a Glasgow Outcome Scale (GOS) 5 (comparable with mRS-score 0–1) was reported in 63% of patients [37]. One possible explanation for the differences in outcomes is our use of a standardized functional assessment protocol. Consequently, the lower proportions of mRS-score 0–1 and 0–2 observed in this study may reflect a more precise, and therefore more reliable, measurement of true functional status. In the present study, an mRS-score 1 was the most common score. This finding may suggest ongoing functional recovery beyond six months, given that the majority of patients reports an mRS-score 2 at six months’ follow-up [10].

Residual symptoms affect post-stroke quality of life [38]. In our cohort, 75% of nSAH patients reported cognitive and/or emotional complaints on the CLCE-24, and only a small proportion denied any symptoms, which is lower than previously reported for nSAH [12]. The most frequently reported complaints were a decreased level of energy, difficulties remembering new information, being more emotional, and difficulties keeping up with people or life in general, similar to patterns seen after aSAH [39]. SSQoL scores revealed greater impairment in the psychosocial than in the physical domain, particularly in energy level and cognition, consistent with the CLCE-24, and overall SSQoL scores (total, physical, psychosocial) were comparable to aSAH populations [37, 40]. These symptoms may have contributed to not fully returning to work. Previous studies in nSAH report full RTW rates ranging from 41 to 87% [20–22, 25]. Krajewski and colleagues found that 57% of PMSAH patients with a mRS 0–1 at 2-year follow-up could not resume their previous profession, a proportion similar to their aSAH cohort [20]. In our cohort, 28% of patients did not (fully) return to work. Since we only assessed outcome in living patients, the expected RTW is worse when considering all patients including those who died between admission after the hemorrhage and follow-up. Although the exact reasons for not fully returning to work remain unclear, and some patients may have retired between discharge and follow-up, the presence of residual symptoms may have influenced the likelihood for not (fully) returning to work [41, 42].

Stratification of nSAH into NPSAH and PMSAH is often done for both clinical and research purposes. A large long-term cohort study reported impairments in QoL in the physical, psychosocial, and social domains in both groups and found that NPSAH patients in particular had worse QoL than the general population [43]. In our cohort, most patients had NPSAH. These patients were older, less likely to be employed, and more likely to develop hydrocephalus than PMSAH patients. Despite this, functional outcome, full RTW, residual symptoms, and experienced QoL did not differ between groups. This is notable given that PMSAH is often described as having a ‘benign clinical course’ and ‘excellent outcome’, which is often by the virtue of comparison with aSAH [44]. These patients still appear to need structured counseling, follow-up, and possible rehabilitation. Nevertheless, most nSAH patients in our study did not receive rehabilitation, especially those with PMSAH.

This study has several strengths and limitations. The multicenter design allowed inclusion of a large cohort, which is to our knowledge one of the largest to date assessing outcomes at least one year after the hemorrhage [6, 12, 13, 19, 43]. The prospective design yielded a reliable dataset with <10% missing data in the included patients, and the use of PROMs, although not validated for SAH specifically but based on expert opinion, and the mRS supports the validity of the findings. Standardized interviews performed by two trained researchers further limited bias. Several imitations should be noted. Firstly, the single follow-up assessment, with variable timing (from one year to several years after the hemorrhage). This may have biased outcomes toward better recovery in patients assessed later and worse recovery in those assessed earlier. This also applies to RTW. Repeated assessment would have better captured recovery trajectories. It may have been the case that certain life events, including mandatory retirement have influenced the presented outcomes. Secondly, the risk for selection bias due to differences between responders and non-responders, particularly for employment status. The response rate of 42%, which is acceptable for a long-term cohort study, still may have introduced selection bias. Thirdly, the use of a control group matched approach (e.g. healthy individuals or patients with mild TBI) may have improved the study and facilitate a clearer interpretation of the presented data. Finally, some differences in baseline demographics, including smoking status, existed between the centers and may have introduced bias.

Future studies should address several points. First, recovery should be assessed at multiple timepoints to better characterize trajectories over time. Second, the present study aimed to explore whether patients reported complaints at long-term follow-up. Future studies should focus on prediction of outcome in certain subsets of patients using regression models. Third, in our cohort, only 45% of patients were referred for rehabilitation, mostly NPSAH patients. This relatively low rate may reflect referral bias driven by the expectation of an ‘excellent outcome’, with only the most severely affected patients being referred for rehabilitation. Although rehabilitation in nSAH has been sparsely studied, available data suggest beneficial short-term effect in both NPSAH and PMSAH patients [23, 45]. Larger prospective studies on rehabilitation are therefore needed.

## Conclusion

This study demonstrates that at long-term follow-up, 40% of patients who experience a non-aneurysmal subarachnoid hemorrhage report persistent impairments that limit their ability to resume previously performed activities; notably, only 28% of those previously employed have fully returned to work, a level of disability exceeding an mRS-score of 1. Residual symptoms are present in the majority of patients. Future studies should identify predictors and confounding factors influencing functional recovery and evaluate possible treatment strategies, such as rehabilitation programs to improve long-term outcomes.

## Supplementary Information

Below is the link to the electronic supplementary material.

Supplementary file1 (DOCX 36 KB)

## Data Availability

All data requests should be submitted to Dagmar Verbaan, PhD (d.verbaan@amsterdamumc.nl) for consideration. Access to anonymized data may be granted following review.
