# Supplementary material for: Long-term outcomes after non-aneurysmal, non-traumatic subarachnoid hemorrhage: a prospective multicenter outcome study
Source: J Neurol. 2026 Jun 25;273(7):420. doi: 10.1007/s00415-026-13939-2 (PMC13303305; doi:10.1007/s00415-026-13939-2)
Supplement: Supplementary file 1 — Supplementary file1 (DOCX 36 KB) [file 415_2026_13939_MOESM1_ESM.docx]

**Supplemental Material**

**Supplement Table 1. Baseline characteristics and clinical (responders versus non-responders)**

|  | **nSAH**  **responders**  n=111 | **nSAH**  **non-responders**  n=151 | **total**  n=262 | *p-value* |
| --- | --- | --- | --- | --- |
| **Age,** years, mean (SD) | 55.6 (9.6) | 54.3 (12.3) | 54.8 (11.2) | 0.346 |
| **Sex**, female | 43 (39) | 73 (48) | 116 (44) | 0.132 |
| **Medical history** |  |  |  |  |
| Hypertension | 27 (24) | 52 (34) | 79 (30) | 0.102 |
| Cardiovascular disease | 10 (9) | 27 (18) | 37 (14) | 0.049 |
| Intracranial hemorrhage | 1 (1) | 1 (1) | 2 (1) | 1.000 |
| Anticoagulation/ -platelet therapy | 10 (9) | 24 (16) | 34 (13) | 0.136 |
| Smoking | 47 (42) | 61 (41) | 108 (41) | 0.801 |
| **Employment status** |  |  |  | 0.017 |
| Employed | 87 (78) | 93 (64) | 180 (70) |  |
| Unemployed | 0 (-) | 4 (3) | 4 (2) |  |
| Not-applicable* | 24 (22) | 49 (34) | 73 (28) |  |
| Unknown | 0 (-) | 5 (3) | 5 (1) |  |
| **Diagnosis** |  |  |  | 0.898 |
| NPSAH | 66 (60) | 92 (61) | 158 (60) |  |
| PMSAH | 45 (41) | 59 (39) | 104 (40) |  |
| **Admission characteristics** |  |  |  |  |
| WFNS grade I-III | 108 (97) | 146 (97) | 254 (97) | 1.000 |
| **Complication** |  |  |  |  |
| Acute hydrocephalus | 25 (23) | 26 (17) | 51 (20) | 0.344 |
| Recurrent bleeding | 0 (-) | 1 (1) | 1 (<1) | 0.390 |
| Delayed cerebral ischemia | 3 (3) | 5 (3) | 8 (3) | 1.000 |
| **Mortality** | 4 | 1 | 5 |  |
| **Rehabilitation** |  |  |  |  |
| No rehabilitation | 61 (55) | 89 (64) | 150 (61) |  |
| Rehabilitation (any form) | 50 (45) | 45 (30) | 95 (36) | 0.087 |
| Clinical | 13 (12) | 18 (12) | 31 (12) |  |
| Ambulatory | 33 (30) | 19 (13) | 52 (20) |  |
| Both | 3 (3) | 2 (1) | 5 (1) |  |
| Form unknown | 0 (-) | 3 (2) | 3 (1) |  |
| Missing | 0 (-) | 17 (6) | 17 (3) |  |

*Table 1. provides an overview and comparison of baseline demographics and clinical course characteristics between responders and non-responders. Data is presented as numbers (%) unless otherwise specified. *Including retired, stay-at-home, and incapacitated patients.*

**Supplement table 2. Comparison of baseline characteristics and clinical course between centers**

|  | **AUMC**  n=84 | **USZ**  n=27 | **Total**  n=111 | *p-value* |
| --- | --- | --- | --- | --- |
| **Age,** years, mean (SD) | 55.4 (9.2) | 56.1 (10.8) | 55.6 (9.6) | 0.740 |
| **Sex**, female | 29 (35) | 14 (52) | 43 (39) | 0.108 |
| **Medical history** |  |  |  |  |
| Hypertension | 19 (23) | 8 (30) | 27 (24) | 0.460 |
| Cardiovascular disease | 10 (12) | 0 (-) | 10 (9) | 0.060 |
| Intracranial hemorrhage | 1 (1) | 0 (-) | 1 (1) | 0.569 |
| Anticoagulation/ -platelet therapy | 9 (11) | 1 (4) | 10 (9) | 0.268 |
| Smoking | 43 (51) | 4 (15) | 47 (42) | <0.001 |
| **Employment status** |  |  |  |  |
| Employed | 66 (79) | 21 (78) | 87 (78) | 0.931 |
| Not-employed* | 18 (21) | 6 (22) | 27 (22) |  |
| **Diagnosis** |  |  |  | 0.981 |
| NPSAH | 50 (60) | 16 (59) | 66 (60) |  |
| PMSAH | 34 (41) | 11 (41) | 45 (41) |  |
| **Admission characteristics** |  |  |  |  |
| WFNS grade I-III | 81 (96) | 27 (100) | 108 (97) | 0.319 |
| **Complication** |  |  |  |  |
| Acute hydrocephalus | 21 (25) | 4 (15) | 25 (23) | 0.270 |
| Recurrent bleeding | 0 (-) | 0 (-) | 0 (-) | Na |
| Delayed cerebral ischemia | 3 (4) | 0 (-) | 3 (3) | 0.319 |
| **Rehabilitation** |  |  |  | 0.088 |
| Rehabilitation (any form) | 34 (41) | 16 (59) | 50 (45) |  |
| Follow-up time, months, median (IQR) | 55.7 (25.9 – 72.3) | 62.3 (45.6 – 74.8) | 58.2 (28.9 – 72.3) | 0.134 |
| **Outcome** |  |  |  |  |
| mRS 0 – 2 † | 74 (90) | 27 (100) | 101 (93) | 0.092 |
| mRS 0 – 1 † | 47 (57) | 18 (67) | 65 (60) | 0.390 |
| Return to work (fully & partially) ‡ | 53 (83) | 19 (91) | 72 (85) | 0.397 |

*Table 1. provides an overview and comparison of baseline demographics and clinical course characteristics between responders from both centers. Data is presented as numbers (%) unless otherwise specified. *^1^Including retired, stay-at-home, and incapacitated patients. †Calculated over 109 (82 [center A], 27 [center B]) patients. ‡Calculated over 85 (64 [center A], 21 [center B]) patients.*

**Supplement table 3. CLCE-24**, scores per domain

| **Domain** | **NPSAH** | **PMSAH** | **nSAH** | **P value** |
| --- | --- | --- | --- | --- |
| *Cognitive domain* |  |  |  |  |
| Doing two things at once | 14 (21) | 7 (16) | 21 (19) | 0.622 |
| Decreased attention | 28 (42) | 19 (42) | 47 (42) | 1.000 |
| Keeping up; has become slower | 30 (46) | 16 (36) | 46 (41) | 0.331 |
| Remembering new information | 34 (52) | 20 (44) | 54 (49) | 0.562 |
| Increased forgetfulness | 26 (39) | 14 (31) | 40 (36) | 0.344 |
| Difficulty taking initiative | 22 (33) | 12 (27) | 34 (31) | 0.532 |
| Difficulty planning and organizing | 23 (35) | 13 (29) | 36 (32) | 0.542 |
| Difficulty executing tasks | 4 (6) | 3 (7) | 7 (6) | 0.221 |
| Decreased perception of time | 7 (11) | 4 (9) | 11 (10) | 1.000 |
| Decreased perception of place or spaces | 2 (3) | 2 (4) | 4 (4) | 1.000 |
| Decreased comprehension of language | 9 (14) | 6 (13) | 15 (26) | 1.000 |
| Difficulty speaking or writing | 18 (27) | 11 (24) | 29 (26) | 0.827 |
| Decreased attention for body or surrounding | 4 (6) | 2 (4) | 6 (5) | 1.000 |
|  |  |  |  |  |
| *Emotional domain* |  |  |  |  |
| Depressed | 15 (23) | 10 (22) | 25 (23) | 1.000 |
| Anxiety | 12 (18) | 7 (16) | 19 (17) | 0.801 |
| Less socially oriented | 17 (26) | 14 (31) | 31 (28) | 0.667 |
| Unrealistic expectations | 6 (9) | 8 (18) | 14 (13) | 0.179 |
| Emotional, crying faster | 25 (38) | 19 (42) | 44 (40) | 0.655 |
| Irritable | 31 (47) | 20 (44) | 51 (46) | 0.848 |
| Indifference | 13 (20) | 5 (11) | 18 (16) | 0.245 |
| Less in control of its own behavior | 9 (14) | 5 (11) | 14 (13) | 0.777 |
| Increased tiredness | 36 (55) | 23 (51) | 59 (53) | 0.847 |

Numbers and percentages (%)

**Supplement table 4. SSQoL**, mean and median scores per subdomain

|  | **NPSAH** | | **PMSAH** | | **nSAH** | |
| --- | --- | --- | --- | --- | --- | --- |
|  | Mean (SD) | Median (IQR) | Mean (SD) | Median (IQR) | Mean (SD) | Median (IQR) |
| Total | 4.40 (0.70) | 4.64 (0.93) | 4.34 (0.76) | 4.73 (1.29) | 4.38 (0.72) | 4.71 (1.03) |
| Physical | 4.61 (0.49) | 4.78 (0.55) | 4.66 (0.49) | 4.89 (0.67) | 4.63 (0.49) | 4.85 (0.61) |
| Psychosocial | 4.09 (0.97) | 4.41 (1.09) | 3.94 (1.18) | 4.55 (2.07) | 4.03 (1.06) | 4.45 (1.45) |
| *Domains* |  |  |  |  |  |  |
| Language | 4.47 (0.76) | 4.80 (0.20) | 4.57 (0.74) | 5.00 (0.60) | 4.51 (0.75) | 4.80 (0.60) |
| Self-care | 4.78 (0.37) | 5.00 (0.20) | 4.72 (0.53) | 5.00 (0.30) | 4.75 (0.44) | 5.00 (0.20) |
| Vision | 4.65 (0.64) | 5.00 (0.58) | 4.78 (0.54) | 5.00 (0.17) | 4.70 (0.60) | 5.00 (0.33) |
| Mobility | 4.55 (0.70) | 5.00 (0.79) | 4.64 (0.74) | 5.00 (0.33) | 4.59 (0.71) | 5.00 (0.50) |
| Work | 4.50 (0.86) | 5.00 (0.92) | 4.32 (1.09) | 5.00 (1.17) | 4.42 (0.96) | 5.00 (1.00) |
| Upper extremity function | 4.71 (0.49) | 5.00 (0.55) | 4.83 (0.36) | 5.00 (0.20) | 4.76 (0.44) | 5.00 (0.30) |
| Thinking | 3.60 (1.27) | 4.00 (2.25) | 3.53 (1.48) | 4.00 (2.83) | 3.57 (1.35) | 4.00 (2.33) |
| Personality | 4.04 (1.16) | 4.50 (1.67) | 3.90 (1.36) | 4.67 (2.33) | 3.98 (1.25) | 4.67 (1.83) |
| Mood | 4.36 (1.00) | 5.00 (1.00) | 4.12 (1.15) | 4.60 (2.00) | 4.26 (1.06) | 5.00 (1.10) |
| Family role | 4.34 (1.02) | 5.00 (1.00) | 4.17 (1.14) | 5.00 (1.50) | 4.27 (1.07) | 5.00 (1.00) |
| Social role | 4.05 (1.11) | 4.50 (1.95) | 3.96 (1.26) | 4.60 (2.00) | 4.01 (1.17) | 4.60 (2.00) |
| Energy | 4.00 (1.27) | 4.67 (2.00) | 3.79 (1.52) | 4.67 (2.50) | 3.91 (1.38) | 4.67 (2.00) |
